# Survival results according to Oncotype Dx recurrence score in patients with hormone receptor positive HER-2 negative early-stage breast cancer: first multicenter Oncotype Dx recurrence score survival data of Turkey

**DOI:** 10.3389/fonc.2023.1151733

**Published:** 2023-06-28

**Authors:** Çağlar Ünal, Tolga Özmen, Çetin Ordu, Kezban Nur Pilanci, Ahmet Serkan İlgün, Erhan Gökmen, Elvina Almuradova, Mustafa Özdoğan, Nilüfer Güler, Cihan Uras, Halil Kara, Orhan Demircan, Selver Işık, Gül Alço, Pınar Saip, Esra Aydın, Tomris Duymaz, Filiz Çelebi, Kanay Yararbaş, Gursel Soybir, Vahit Ozmen

**Affiliations:** ^1^ Division of Medical Oncology, Department of Internal Medicine, Kartal Dr. Lütfi Kırdar City Hospital, İstanbul, Türkiye; ^2^ Division of Gastrointestinal and Oncologic Surgery, Harvard Medical School, Boston, MA, United States; ^3^ Division of Gastrointestinal and Oncologic Surgery, Massachusetts General Hospital, Boston, MA, United States; ^4^ Division of Medical Oncology, Department of Internal Medicine, Gayrettepe Florence Nightingale Hospital, İstanbul, Türkiye; ^5^ Division of Medical Oncology, Department of Internal Medicine, Memorial Bahçelievler Hospital, İstanbul, Türkiye; ^6^ Department of Surgery, Mater Dei Hospital, Msida, Malta; ^7^ Division of Medical Oncology, Department of Internal Medicine, Ege University School of Medicine, Izmir, Türkiye; ^8^ Division of Medical Oncology, Department of Internal Medicine, Tınaztepe Galen Bayraklı Hospital, Izmir, Türkiye; ^9^ Division of Medical Oncology, Department of Internal Medicine, Akdeniz University, Antalya, Türkiye; ^10^ Division of Medical Oncology, Department of Internal Medicine, Hacettepe University Institute of Oncology, Ankara, Türkiye; ^11^ Department of General Surgery, Acıbadem University, İstanbul, Türkiye; ^12^ Department of General Surgery, Çukurova University School of Medicine, Adana, Türkiye; ^13^ Division of Medical Oncology, Department of Internal Medicine, Marmara University Hospital, İstanbul, Türkiye; ^14^ Department of Radiation Oncology, Gayrettepe Florence Nightingale Hospital, İstanbul, Türkiye; ^15^ Division of Medical Oncology, Department of Internal Medicine, İstanbul University Institute of Oncology, İstanbul, Türkiye; ^16^ Department of Physiotherapy and Rehabilitation, Faculty of Health Sciences, İstanbul Bilgi University, İstanbul, Türkiye; ^17^ Department of Radiology, Yeditepe University Hospital, İstanbul, Türkiye; ^18^ Department of Medical Genetics, Demiroglu Bilim University, İstanbul, Türkiye; ^19^ Department of General Surgery, Memorial Şişli Hospital, Istanbul, Türkiye; ^20^ Department of General Surgery, İstanbul University İstanbul School of Medicine, İstanbul, Türkiye

**Keywords:** clinicopathologic characteristics, early-stage breast cancer, lymph node-negative, Oncotype DX^®^, recurrence score, 21 genes

## Abstract

**Background:**

The Oncotype Dx recurrence score (ODx-RS) guides the adjuvant chemotherapy decision-making process for patients with early-stage hormone receptor-positive, HER-2 receptor-negative breast cancer. This study aimed to evaluate survival and its correlation with ODx-RS in pT1-2, N0-N1mic patients treated with adjuvant therapy based on tumor board decisions.

**Patients and methods:**

Estrogen-positive HER-2 negative early-stage breast cancer patients (pT1-2 N0, N1mic) with known ODx-RS, operated on between 2010 and 2014, were included in this study. The primary aim was to evaluate 5-year disease-free survival (DFS) rates according to ODX-RS.

**Results:**

A total of 203 eligible patients were included in the study, with a median age of 48 (range 26-75) and median follow-up of 84 (range 23-138) months. ROC curve analysis for all patients revealed a recurrence cut-off age of 45 years, prompting evaluation by grouping patients as ≤45 years vs. >45 years. No significant difference in five-year DFS rates was observed between the endocrine-only (ET) and chemo-endocrine (CE) groups. However, among the ET group, DFS was higher in patients over 45 years compared to those aged ≤45 years. When stratifying by ODx-RS as 0-17 and ≥18, DFS was significantly higher in the former group within the ET group. However, such differences were not seen in the CE group. In the ET group, an ODx-RS ≥18 and menopausal status were identified as independent factors affecting survival, with only an ODx-RS ≥18 impacting DFS in patients aged ≤45 years. The ROC curve analysis for this subgroup found the ODx-RS cut-off to be 18.

**Conclusion:**

This first multicenter Oncotype Dx survival analysis in Turkey demonstrates the importance of Oncotype Dx recurrence score and age in determining treatment strategies for early-stage breast cancer patients. As a different aproach to the literature, our findings suggest that the addition of chemotherapy to endocrine therapy in young patients (≤45 years) with Oncotype Dx recurrence scores of ≥18 improves DFS.

## Introduction

Breast cancer has now surpassed lung cancer as the most common cancer worldwide, accounting for 2.3 million new cases each year (1). Particularly in Turkey, breast cancer is the most prevalent cancer in women, constituting 24,175 cases (23.9%) in 2020 (2). Notably, a significant proportion of new patients, 27% and 45% respectively, were diagnosed at stages 1 and 2 (3). A majority of early-stage breast cancer patients, approximately 70%, present with hormone receptor (HR) positive and HER-2 negative profiles (4).

While adjuvant chemotherapy can decrease cancer-related mortality by 5-15% (5), its benefits for early-stage breast cancer patients (ER+, HER-2 -, pN0) remain contentious (6). Many studies have proposed that a substantial fraction of these patients may not require adjuvant systemic treatment (7, 8). Conversely, other research has indicated that adjuvant chemotherapy can decrease mortality rates by 1-5% in patients with early-stage hormone receptor positive lymph node negative breast cancer (5, 9).

Recently, the use of genomic tests, which aid in determining the efficacy of systemic chemotherapy, has increased (10). The prognostic and predictive value of the Oncotype Dx (ODx) test (Genomic Health, Redwood City, CA, USA), which evaluates 21 genes, has been validated for patients with HR positive, HER-2 negative, and lymph node-negative breast cancer (11–14). Endorsed by the American Society of Clinical Oncology (ASCO), the National Comprehensive Cancer Network (NCCN), and other guidelines (11, 13), the ODx Recurrence Score (ODx-RS) test is utilized to gauge the advantage of adjuvant chemotherapy. Based on the ODx-RS, patients are categorized into three groups: low risk (RS<18), medium risk (RS 18-30), and high risk (RS>30), with their respective risks of distant recurrence at 6.8%, 14.3%, and 30.5% (15). While the low-risk group sees no benefit from chemotherapy, it is evidently beneficial for the high-risk group. The advantage for the intermediate-risk group, however, remains unclear (16).

Younger patients tend to have a higher risk of breast cancer recurrence and a lower survival rate compared to older patients (17). In the USA, 19% (48,080) of patients diagnosed with breast cancer are women under 50 years of age (18). In contrast, nearly 50% of patients in our breast cancer registry in Turkey were under the age of 50 due to the younger population structure (19). Given the more aggressive biological behavior of the tumor and distinct clinical features in young patients, this group warrants closer examination (20). Adjuvant chemotherapy has been shown to significantly reduce the risk of recurrence in young women, and the beneficial effects of adjuvant endocrine therapy on survival in hormone receptor positive patients are also recognized (21).

The aim of our study is to investigate the factors influencing recurrence in HR positive and HER-2 negative patients who have undergone surgery for early-stage breast cancer, and to identify the correlation between ODx-RS and disease-free survival (DFS) in Turkish breast cancer patients.

## Methods

### Study design and participants

A retrospective analysis was performed on all patients who had Oncotype Dx risk scores (ODx-RS) across ten medical centers between 2010 and 2014. From this group, 18 patients were excluded due to irregular follow-up visits, thus leaving us unable to obtain their final status. The study eventually included a total of 203 women diagnosed with hormone receptor positive, HER-2 negative early-stage breast cancer (pT1-2, pN0-N1mic, M0). These patients were treated in ten different hospitals across Turkey within the same timeframe and had ODx-RS assessments to inform the decision for chemotherapy.

Patient demographic, clinical, and pathological details including age, tumor size, histological grade, Estrogen receptor (ER) and Progesterone receptor (PR) status, Ki67 index, and lymph node status were recorded retrospectively. The ODx-RS was examined using tissue sections taken from surgically removed, formalin-fixed, paraffin-embedded samples in a centralized laboratory. If nuclear staining was moderate to strong in at least 1% of tumor cells upon immunohistochemical testing, ER and/or PR were considered positive. HER-2 expression was evaluated using immunohistochemical (IHC) staining. A score of 0 or 1 on the IHC staining was interpreted as negative for HER-2. In cases where the IHC score was 2, further assessment was conducted using a Fluorescence *In Situ* Hybridization (FISH) test. Only those with a negative FISH test result were included in the study. Patients were classified based on the clinical risk associated with their tumors. Clinical risk was categorized into two levels. ‘Low-risk’ classification was given under these conditions: a low-grade tumor up to 3 cm, an intermediate-grade tumor up to 2 cm, or a high-grade tumor up to 1 cm in size. If a tumor did not fit into any of these categories, it was considered ‘high-risk (22). Patients were divided into two groups according to ODx-RS: 0-17 and ≥18. An oncotype score cut-off value of 18 for chemotherapy administration was used, based on the NSABP-20 study (23). Our research aimed to remove uncertainty in treatment decisions by dividing patients into two groups: 0-17 and ≥18.

Even with the known ODx scores, the choice of adjuvant therapy was determined in weekly tumor board meetings. Patients were split into two categories: those who received hormone therapy alone and those who received chemotherapy (taxane-based and/or adriamycin-based regimens) in combination with hormone therapy (tamoxifen or aromatase inhibitors ± LHRH analog). The primary aim was the five-year Disease-Free Survival (DFS) rate, with DFS being defined as the period from treatment to local, distant disease recurrence or death from any cause.

Approval was granted by the Ethics Committee of Istanbul Bilgi University (Project number: 2022-40034-118).

### Statistical analysis

Categorical values such as demographic and clinical characteristics were compared using the chi-square test. Descriptive statistical analysis was used to evaluate age across groups by considering median, lowest, and highest values. Five-year DFS values were evaluated with Kaplan-Meier analysis, and independent prognostic factors affecting DFS were identified using multivariate Cox regression. Receiver Operating Characteristics (ROC) curve analysis was conducted to determine the cut-off for adding chemotherapy. All statistical analyses were performed using SPSS 22.0, and a p-value <0.05 was considered statistically significant.

## Results

The median age of patients was 48 years (ranging from 26 to 75), and the median follow-up period was 84 months (ranging from 23 to 138). The median Oncotype Dx risk score (ODx-RS) was 16 (ranging from 0 to 58). All patients (n=203,100%) were diagnosed with ER-positive breast cancer, and 173 (85.2%) were PR-positive. There were a total of 14 recurrences (6.89%), with 5 local recurrences (2.46%) and 9 distant recurrences (4.4%). Two patients died due to unrelated causes.

**Figure 1:**
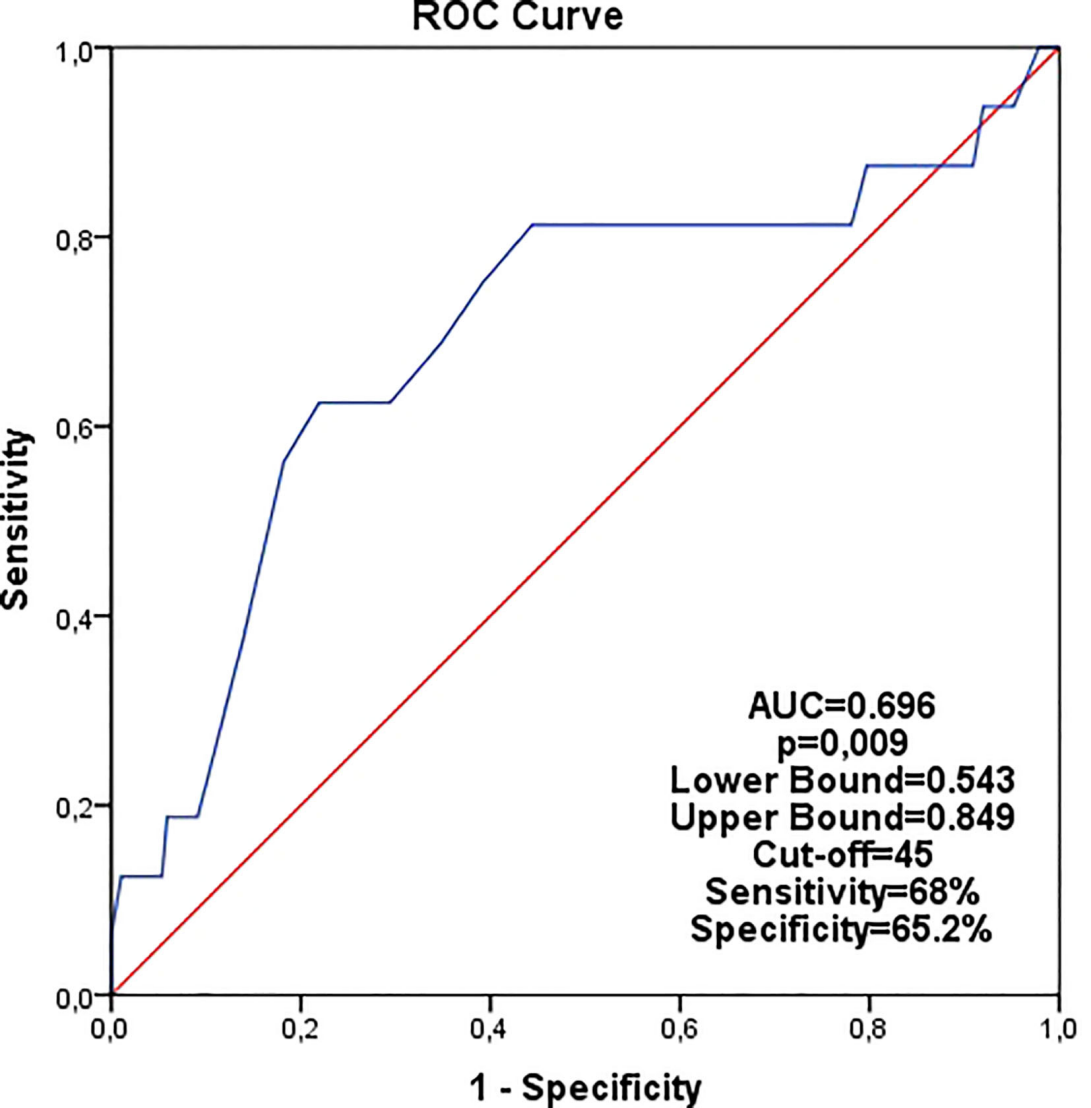
Analysis of age in the all patients for recurrence by ROC curve (cutoff age was found 45).

In the ROC curve analysis for recurrence among all patients, the age cut-off was determined as 45 years (**Figure 1**). Consequently, patients were divided into two groups: ≤45 years and >45 years.

Seventy-four patients (36.5%) were aged ≤ 45 years. Endocrine therapy alone was administered to 146 (71.9%) patients, while 57 patients (28.1%) received a combination of systemic chemotherapy and endocrine therapy (CT+ET). The ODx scores ranged from 0-17 in 117 patients (57.6%), 18-30 in 69 patients (34%), and over 30 in 17 patients (8.4%) (**Table 1**).

**Table 1:** Characteristics of the patients at baseline.

	All patients	ET	CT+ET	p value^#^
Age ≤45yr	74 (36.5%)	52 (35.6%)	22 (38.6%)	0,69
Age >45 yr	129 (63.5%)	94 (64.4%)	35 (61.4%)	
**The pT stage**				
pT1	133 (65.5%)	106 (72.6%)	27 (47.4%)	**0,001**
pT2	70 (34.5%)	40 (27.4%)	30 (52.6%)	
**The Histological subtype**				
IDC	153 (75.4%)	107 (73.3%)	46 (80.7%)	0,27
Other subtypes*	50 (24.6%)	39 (26.7%)	11 (19.3%)	
**Menopausal status**				
Premenopausal	111 (54.7%)	75 (51.4%)	36 ( 63.2%)	0,12
Postmenopausal	92 (45.3%)	71 (48.6%)	21 (36.8%)	
**Histologic grade**				
Grade 1	28 (13.8%)	25 (17.1%)	3 (5.3%)	**0,002**
Grade 2	138 (63.0%)	102 (69.9%)	36 (63.2%)	
Grade 3	37 (18.2%)	19 (13.0%)	18 (31.6%)	
**PR receptor status**				
Positive	173 (85.2%)	130 (89.0%)	43 (75.4%)	**0,01**
Negative	30 (14.8%)	16 (11.0%)	14 (24.6%)	
**Clinical risk score**				
Low	111 (54.7%)	92 (63.0%)	19 (33.3%)	**<0,001**
High	92 (45.3%)	54 (37.0%)	38 (66.7%)	
**The pathologic stage**				
Stage 1a	175 (86.2%)	134 (91.8%)	41 (71.9%)	**<0,001**
Stage 1b	18 (8.9%)	11 (7.5%)	7 (12.3%)	
Stage 2a	10 (4.9%)	1 (0.7%)	9 (15.8%)	
**Lymphatic invasion **				
Negative	143 (73.7%)	109 (77.3%)	34 (64.2%)	0,06
Positive	51 (26.3%)	32 (22.7%)	19 (35.8%)	
**Vascular invasion**				
Negative	156 (80.4%)	120 (85.1%)	36 (67.9%)	**0,007**
Positive	38 (19.6%)	21 (14.9%)	17 (32.1%)	
**Oncotype score<18 vs Oncotype≥18**				
Oncotype RS<18	117 (57.6%)	110 (75.3%)	7 (12.2%)	**<0,001**
Oncotype RS≥18	86 (34%)	36 (24.7%)	50 (87.8%)	
**The pN stage**				
pN0	191 (94.1%)	140 (95.9%)	49 (89.5%)	0,08
pN1mic	12 (5.9%)	6 (4.1%)	6 (10.5%)	
**Ki67 status**				
Ki67<20	94 (46.3%)	74 (50.7%)	20 (35.1%)	**0,04**
Ki67≥20	67 (33%)	41 (28.1%)	26 (45.6%)	
Missing	42 (20.7%)	31 (21.2%)	11 (19.3%)	
**Type of axillary surgery**				
Axillary dissection	31 (17.7%)	22 (17.8%)	9 (17.5%)	0,89
SLNB †	172 (82.3%)	124 (82.2%)	48 (82.5%)	
**Traditional recurrence risk categories**				
0-17	117 (57.6%)	110 (75.3%)	7 (12.2%)	**<0,001**
18-30	69 (34%)	35 (23.9%)	34 (59.6%)	
>30	17 (8.4%)	1 (0.6%)	16 (28.2%)	

All the values presented as n(%).

#chi square test, IDC, invasive ductal carcinoma. ET, endocrine therapy; CT, chemotherapy.

*invasive lobuler carcinoma, mucinous, metaplastic, micropapiller, cribriform, papiller †sentinel lymph node biopsy. P-values that are less than 0.05 are accentuated in bold within the table.

There was no significant difference in Disease-Free Survival (DFS) rates when using 50 years as the age threshold (DFS: 92.3% vs. 97.7%, p=0.107). However, patients older than 45 years demonstrated significantly better DFS than those aged 45 years or younger (DFS: 98.4% vs. 89.2%, p=0.009, HR:3.62, 95% CI:1.28-10.1; p=0.015).

There was no significant difference in DFS between the endocrine-only group and the chemo-endocrine group (93% vs. 95.9% respectively, p=0.14). The analysis of menopausal status revealed significantly higher DFS in postmenopausal patients in both the overall cohort and in the endocrine-only group (five-year DFS rates: premenopausal 91.9%, postmenopausal 97.8%, p=0.01, all groups; premenopausal 92.0%, postmenopausal 98.6%, p=0.01, endocrine-only group) (**Table 2**).

**Table 2:** Evaluation of DFS by Kaplan-Meier analysis.

All Patients	Five-year rates of DFS(%)	p value
**Age ≤45yr**	89.2	**0,009**
**Age >45 yr**	98.4	
**ET**	93.0	0,14
**CT+ET**	95.9	
**Premenopausal**	91.9	**0,01**
**Postmenopausal**	97.8	
**Oncotype<18**	98.4	**0,001**
**Oncotype≥18**	88.2	
Endocrine-Only Group		
**Age ≤45yr**	90.4	**0,02**
**Age >45 yr**	98.9	
**Premenopausal**	92.0	**0,01**
**Postmenopausal**	98.6	
**Oncotype<18**	98.2	**0,01**
**Oncotype≥18**	83.3	
Chemo-endocrine Group*		
**Age ≤45yr**	87.4	0,26
**Age >45 yr**	93.1	
**Premenopausal**	91.7	0,67
**Postmenopausal**	95.2	

*DFS could not be evaluated because there was no recurrence in patients with an ODx<18 in the chemoendocrine group. ET, endocrine therapy; CT, chemotherapy. P-values that are less than 0.05 are accentuated in bold within the table.

**Figure 2:**
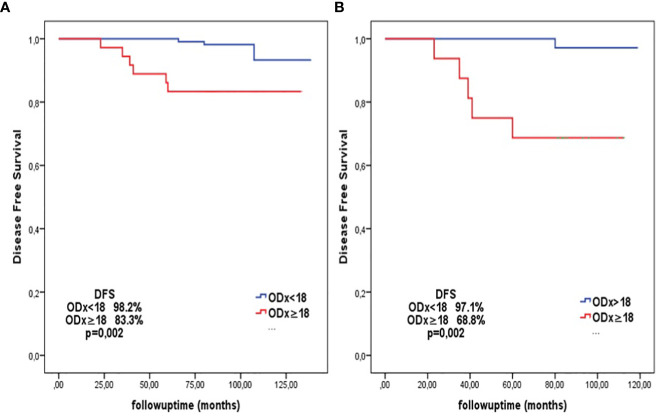
**(A)** (left): DFS analysis according to the cut-off ODx score of 18 in patients in the all endocrine-only group (For ODx score<18 DFS: 98.2%; for ODx score ≥18 DFS: 83.3%, p=0.002). **(B)** (right): DFS analysis according to the cut-off ODx score of 18 in patients aged ≤45 years in the endocrine-only group (For ODx score<18 DFS: 97.1%; for ODx score ≥18 DFS: 68.8%, p=0.002).

In the chemo-endocrine group, DFS was similar between patients aged ≤45 years and those >45 years (87.4% vs 93.1%, p=0.26). In contrast, the endocrine-only group exhibited higher DFS in patients >45 years compared to those ≤45 years (98.9% vs. 90.4%, p=0.024). When dividing patients based on the ODx-RS as 0-17 and ≥18, the former group had significantly higher DFS (98.2% vs 83.3%, p=0.005) (**Figure 2A**). In patients ≤45 years in the endocrine-only group, those with an ODx score ≥18 showed significantly lower DFS compared to those with an ODx score <18 (68.8% vs 97.1%, p=0.002) (**Figure 2B**).

Age (≤45 vs *>*45), ODx score (<18 vs ≥18), and menopausal status were factors influencing DFS in the univariate analysis of the endocrine-only group. Multivariate analysis revealed independent effects of ODx score (<18 vs ≥18) and menopausal status on DFS (ODx score (<18 vs ≥18) HR:8.15, 95% CI: 2.01-32.9, p=0.003; premenopausal vs. postmenopausal HR:8.24, 95% CI: 1.02-66.1; p=0.04) (**Table 3**).

**Table 3:** Factors affecting DFS in the endocrine-only group.

	Univariate analysis			Multivariate analysis		
	HR	95% CI Lower-Upper	p value	HR	95% CI Lower-Upper	p value
**Age (≤45 vs >45)**	4.32	1.06-17.6	**0,04**	1.09	0.21-5.64	0,91
**Oncotype score (<18 vs ≥18)**	9.32	2.32-37.5	**0,002**	6.83	1.70-27.3	**0,007**
**Oncotype score**	1.14	1.04-1.24	**0,03**	1.07	0.88-1.29	0,47
**Premenopausal vs Postmenopausal**	8.35	1.04-66.9	**0,04**	8.24	1.02-66.1	**0,04**
**Age**	0.93	0.86-1.15	0,11			
**PR > 20**	2.07	0.51-8.30	0,30			
**Tumor size**	0.93	0.84-1.04	0,22			
**Histologic grade 1 vs 2-3**	1.73	0.21-13.9	0,60			
**Stage 1 vs Stage 2**	0.04	0- 256	0,57			
**Clinical risk score (low vs high)**	0.51	0.10-2.46	0,40			
**Lymphatic invasion**	0.03	0-27.3	0,32			
**Vascular invasion**	0.03	0-146	0,42			

P-values that are less than 0.05 are accentuated in bold within the table.

In patients aged ≤45 years in the endocrine-only group, only the ODx score (<18 vs ≥18) was found to influence DFS (HR:13.4, 95% CI: 1.56-115; p=0.01) (**Table 4**).

**Table 4:** Factors affecting DFS in patients aged ≤45 years in the endocrine-only group*(subgroup analyses).

	HR	95% CI Lower-Upper	p value
**Oncotype score (<18 vs ≥18)**	13.4	1.56-115	**0,01**
**Oncotype score**	1.21	1.04-1.40	**0,01**
**Age**	0.99	0.80-1.23	0,96
**PR > 20**	1.32	0.15-11.3	0,80
**Tumor size**	0.97	0.86-1.08	0,58
**Histologic grade (1vs 2-3)**	1.43	0.16-12.3	0,74
**Stage 1 vs Stage 2**	0.41	0-1806	0,55
**Clinical risk score (low vs high)**	0.89	0.16-4.89	0,90
**Lymphatic invasion**	0.26	0-31.2	0,31
**Vascular invasion**	0.039	0-146	0,32

* Insufficient number of postmenopausal patients under the age of 45 to assess menopausal status. P-values that are less than 0.05 are accentuated in bold within the table.

**Figure 3:**
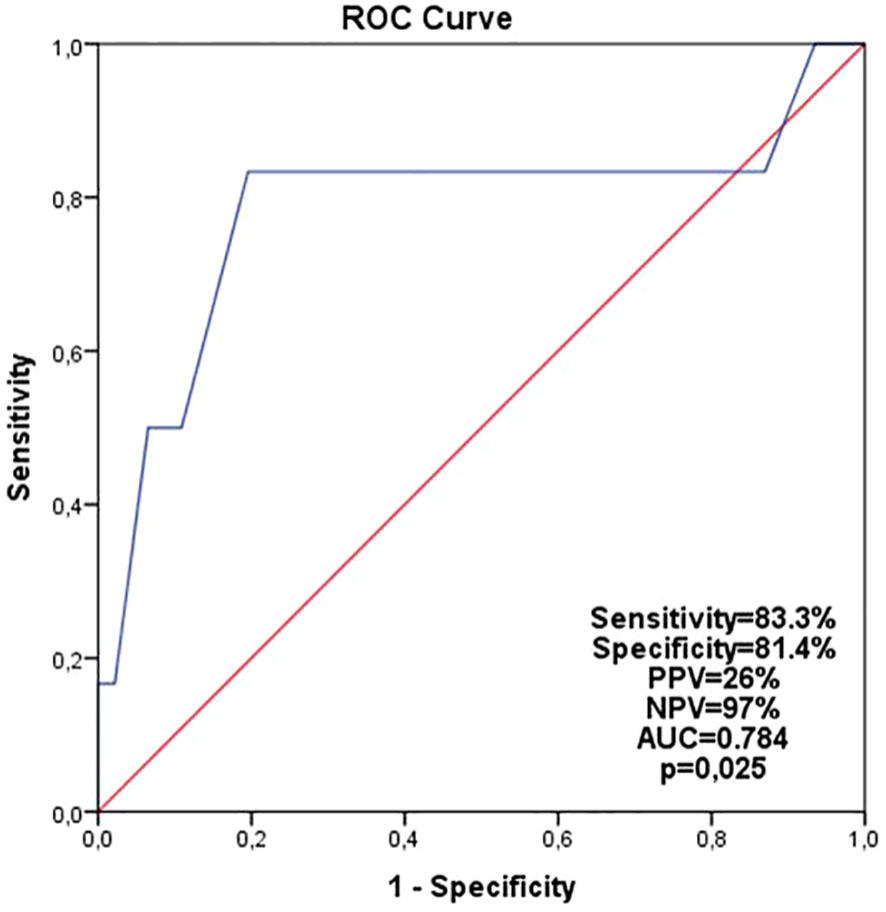
Analysis of patients ≤ 45 years of age in the endocrine-only group by ROC curve (cutoff ODx score was found 18).

In the ROC curve analysis for patients aged ≤45 years in the endocrine-only group, the ODx cut-off score for predicting recurrence was determined as 18. The sensitivity and specificity for this cut-off were 83.3% and 81.4, respectively, with a positive predictive value of 26% and a negative predictive value of 97% (p=0.025) (**Figure 3**).

## Discussion

While the NCCN guidelines do not specify an age cut-off, the ASCO guidelines have set an age cut-off of 50 years based on the Tailor X study. In these studies, patients were divided into two groups, and treatment modalities were arranged according to this age limit of 50 (24–26). This practice is attributed to the typically poorer prognosis of younger patients and the fact that chemotherapy primarily reduces the risk of recurrence due to ovarian suppression (20, 27–29). Furthermore, younger patients often have fewer comorbid diseases, thus demonstrating better tolerance for chemotherapy (20, 28, 29). In contrast to the prevailing literature, we found no significant difference in disease-free survival (DFS) between groups separated by the age of 50 (DFS: 92.3% vs. 97.7%, p=0.107). However, a significant survival difference was observed between groups separated by the age of 45 (DFS 89.2% vs 98.4%, p=0.009). This difference is thought to be attributable to the lower proportion of young patients in the MINDACT and Tailor X studies, the main reference studies, where the majority of the population comprised patients over 50 years of age (21, 22). The EORTC 10041/BIG 03-04 MINDACT study, which used the ≤45 age cut-off for categorizing patients, reported that the tumor biological features of patients in this age group were more aggressive than those in other groups (30). In our ROC curve analysis of all patients for recurrence, the cut-off age was 45 years (**Figure 1**). Consequently, patients were evaluated by dividing them into two groups, ≤45 years and >45 years. Another reason for this adjustment is the notably higher rate of breast cancer patients in Turkey in younger populations compared to Western countries. In a study we conducted with 20,000 breast cancer patients in Turkey, the rate of patients under the age of 40 was 16.5% (19), whereas an analysis of SEER data showed that the prevalence of patients under the age of 40 was merely 1.1% (31). Additionally, the Tailor X study determined that the age most benefitting from chemotherapy was 45 years old, and it also indicated that the benefit of chemotherapy diminishes as age exceeds 45 years (21). In our study, we found a significant DFS difference between patients aged ≤45 years and patients aged >45 years in the endocrine group, while this difference was not observed in the chemo-endocrine group (**Table 2**). These findings underscore the importance of adding chemotherapy to endocrine therapy in young and selected patient groups, as corroborated by the literature.

When we planned the ODx cut-off value as 18 in patients ≤45 years in the ET subgroup, we observed that the DFS rate of patients with an ODx-RS of 18 or above decreased significantly (**Figure 2**). Since the patients in this subgroup had the lowest DFS rates, the cut-off value was determined as 18 in the ROC curve analysis for this patient population (**Figure 3**). An ODx-RS ≥18 in the endocrine-only subgroup aged 45 years and younger was identified as an independent risk factor affecting DFS (**Table 4**). It was determined that the addition of chemotherapy to endocrine therapy was beneficial in patients aged ≤45 years with an ODx score of 18 or above, and the addition of chemotherapy to patients with an ODx-RS of less than 18 did not benefit due to the very high negative predictive value (97%). In the Eastern Cooperative Oncology Group E2197 phase III study, in patients younger than 50 years of age, the 10-year risk of recurrence was in the low-risk (Odx-RS<18) group; it has been observed that the risk of recurrence is significantly lower than the patients with ODx-RS 18-30 and >30 (32) [% (95% CI): 1.9 (0.5–7.9), 8.1 (3.4–19.6), 10.3 (5.4–19.7), p=0.17) respectively]. In the NSABP-14 study, which included only endocrine therapy patients, distant recurrence was observed at a rate of 6.8% in patients with RS below 18, 14.3% in patients with 18-30, and 30.5% in patients over 30 (33). In this study, it was observed that the recurrence rate was significantly lower in patients with ODX-RS < 18 than in patients over 18 (33). Park et al. concluded that there was a 9% increase in the risk of death from breast cancer for each unit increase in ODx-RS in patients with an RS between 18 and 30 (34). In our study, it was determined that each unit increase in the oncotype score in women aged ≤45 years in the endocrine group increased the risk of recurrence by 1.2 times (HR:1.21, 95%Cl; 1.04-1.40, p=0.012) (**Table 4**).

In our study, we found DFS to be statistically significantly lower in premenopausal patients compared to postmenopausal patients, across all patients and in the endocrine-only group. Interestingly, no significant difference was identified in the chemo-endocrine group (**Table 2**). The reason for not detecting a significant survival difference in patients in the chemo-endocrine group is likely due to the contribution of chemotherapy-induced ovarian suppression observed in premenopausal patients, as reported in the Tamoxifen and Exemestane Trial and the Suppression of Ovarian Function Trial (SOFT and TEXT) (35–37). There are numerous studies investigating the relationship between the ODx-RS and certain clinicopathological features (15, 38, 39). In our previous prospective clinical study, we discovered a negative correlation between PR, Ki67 level, and ODx-RS (40). In our study, an ODx RS≥18 (HR: 6.83, 95% CI: 1.70-27.3; p=0.007) and menopause status (HR: 8.24, 95% CI: 1.02-66.1; p=0.04) were identified as independently affecting DFS. However, no relationship was found between DFS and histological grade, PR negativity, clinical risk score, tumor diameter, pathological stage, lymphatic invasion, or vascular invasion (**Table 3**).

This study has several limitations. It’s a retrospective study with a small cohort size from multiple institutions, which necessitates further studies with larger sample sizes. Also, this cohort is predominantly composed of an ethnic minority patient population, which may affect the generalizability of the results. Despite being the largest national study within our country, reaching more patients could help us draw clearer conclusions that are more reflective of the Turkish population, considering our ever-growing population size and the genetic/ethnic variability in our population. Finally, the follow-up time was short, and events were too scarce to perform a stratified analysis. Therefore, more detailed data with a longer follow-up time on a larger multicenter scale are encouraged to evaluate whether early-stage breast cancer patients can be exempted from chemotherapy. A study with a much larger number of patients should be conducted in our country, especially for postmenopausal patients.

In contrast to the Tailor X study, which examined the ODx-RS of 0-10, 11-26, and >26 by dividing patients into three groups (21), in our study, we aimed to organize the treatment modality so that there was no group in which the treatment decision was uncertain by dividing the patient group into 0-17 and ≥18.

Our study is the first multicenter Oncotype Dx survival analysis in Turkey. This study demonstrates that the Oncotype Dx recurrence score and age are crucial factors in making treatment decisions for patients diagnosed with early-stage breast cancer. A study with a much larger number of patients is necessary in our country, especially for postmenopausal patients. In conclusion, our study has shown that adding chemotherapy to endocrine therapy in young (≤45 years) patients with Oncotype Dx recurrence scores of 18 and above contributes to DFS.

## Data availability statement

The raw data supporting the conclusions of this article will be made available by the authors, without undue reservation.

## Ethics statement

This study was performed in line with the principles of the Declaration of Helsinki. Approval was granted by the Ethics Committee of Istanbul Bilgi University(Project number: 2022-40034-118).

## Author contributions

All authors have made a significant contribution to this manuscript, including the design of the study, data registration, statistics, manuscript writing. All authors contributed to the article and approved the submitted version.
